# Are surfers and scuba divers an overlooked at-risk group for age-related macular degeneration?

**DOI:** 10.1038/s41433-025-03941-9

**Published:** 2025-07-21

**Authors:** Davinia Beaver, Carly Hudson

**Affiliations:** 1 Clem Jones Centre for Regenerative Medicine, Gold Coast, QLD Australia; 2 Bond University, Faculty of Health Sciences and Medicine, Gold Coast, QLD Australia; 3 Bond University, Bond Business School, Gold Coast, QLD Australia; 4 University of New England, Faculty of Medicine and Health, Armidale, NSW Australia

**Keywords:** Risk factors, Physiology

## Introduction

Every day, across the world’s coastlines, millions of individuals enter the ocean in pursuit of recreation, connection, and challenge. From surfers waiting in stillness for the next wave to divers submerged just beneath the glittering surface, these individuals are uniquely positioned at the intersection of two environmental factors, sunlight and seawater, that may, in combination and over time, adversely affect ocular health.

There is some literature on the adverse effects of ultraviolet (UV) radiation and blue light (BL) on ocular tissues. These range from pterygium and photokeratitis to age-related macular degeneration (AMD) [1–3]. UV radiation encompasses wavelengths from 100–400 nm, categorised into UVC (100–280 nm), UVB (280–315 nm), and UVA (315–400 nm), while BL occupies the high-energy (HEV) portion of the visible spectrum between 400–500 nm [4]. Research has established risk to ocular health in outdoor workers, welders, and those living in high UV-index regions, affecting the anterior part of the eye and potentially the retina [5–7]. However, populations whose exposure is mediated through water (surfers, scuba divers), remain underrepresented in ophthalmic research.

Surfing and scuba diving are experiencing a global rise in participation as key aspects of coastal lifestyles [8, 9]. These activities offer significant physical and psychological benefits, yet expose participants to prolonged sunlight exposure at the ocean’s surface [10]. While the dermatological consequences of UV radiation are acknowledged, the ocular risks associated with cumulative UV and BL exposure during these sports remain markedly understudied.

Sunlight at sea behaves differently from on land. The ocean’s surface can reflect between 10 and 30% of incident UV radiation, and even more in the case of HEV light under certain angles of incidence and atmospheric conditions [11]. The refractive properties of water can direct upward-scattered light toward the anterior surface of the eye, increasing the irradiance received by the cornea and lens. For surfers who spend hours gazing toward the horizon, and divers who spend extended durations beneath the surface where BL penetrates depths (<40 m), the cumulative exposure may not be trivial. However, water sport participants, particularly surfers and divers, are largely absent from clinical or epidemiological studies investigating light-induced ocular pathology.

The potential consequences of cumulative exposure to UV and BL are biologically plausible and clinically relevant. UV-B radiation has been implicated in pterygium and photokeratitis [12], while both UV-A and BL are increasingly associated with cataractogenesis and AMD [13, 14]. These risks may be amplified in individuals who engage in prolonged surface-level exposure without adequate eye protection, particularly in high UV-index regions such as Australia, Hawaii and Southeast Asia.

Efforts are needed to understand how dynamic outdoor environments shape ocular health and to safeguard the vision of a growing global community. Here, we argue that the lack of ocular research with surfers and divers represents a potential blind spot in our understanding of the environmental impact on eye health. Interdisciplinary efforts to integrate ophthalmology, environmental optics, and sports medicine are warranted to evaluate the nature and magnitude of risk in this population. Empirical insight is needed to inform both clinical surveillance and the development of targeted protective interventions, such as UV/BL-filtering masks, surf-appropriate eyewear, and/or behavioural recommendations.

## A refined search strategy that revealed an absence

We recently conducted a scoping review to synthesise the available literature on ocular UV and BL exposure in water sport participants. Our search strategy (Appendix A) spanned multiple databases and incorporated keywords and mesh terms related to ocular radiation exposure, marine activity, and retinal damage. Despite a comprehensive and iterative refinement of terms, no eligible human studies were found (Appendix B).

## Why this gap matters

Unlike participants in most outdoor pursuits, surfers and divers face dual exposure mechanisms. Firstly, direct solar radiation at the surface, intensified by the reflective properties of the ocean, and secondly, subsurface BL exposure during diving, where UV is largely filtered but BL penetrate deeply and may contribute to cumulative retinal stress. Scuba divers spend hours in environments where BL dominates their visual field, the majority wearing equipment unable to filter BL. Surfers may receive higher UV exposure due to both duration and angle of incidence, with the ocean acting as a mirror.

## Physiological relevance

The lens is particularly susceptible to damage from UV radiation reflected off surfaces like water, sand, and snow [15]. While UVB is mostly absorbed by the cornea and lens, UVA and visible BL can reach the retina [16].

Chronic exposure to HEV light has been implicated in oxidative stress, photoreceptor damage, and retinal pigment epithelium dysfunction [17, 18]. This is particularly relevant for individuals with minimal ocular protection and in areas with prolonged exposure in high UV-index zones.

## Call to Action

We hypothesise that the paucity of studies may stem from difficulties in quantifying ocular irradiance in aquatic environments, a lack of interdisciplinary collaboration between ophthalmologists, ocular scientists and marine or sports health researchers, and less interest in light exposure in recreational versus occupational settings. Despite these challenges, wearable radiometry and ocular imaging tools now offer practical methods that could help to address these gaps.

Therefore, we propose the following directions for future research:

1. Develop innovative tools to measure ocular irradiance in water sport participants.
2. Observational studies to assess degenerative eye conditions like AMD across age groups in water sport participants.
3. Observational studies to identify the correlates and determinants of ocular conditions in young, middle-aged and older water sport participants, including large-scale surveys and secondary analyses of existing health survey data.
4. Retinal imaging studies comparing water sport participants to non-exposed controls, assessing cumulative exposure risk by age.
  If these studies confirm the hypothesis that UV and BL are implicated in a range of optical disorders among water sport participants, then intervention trials to evaluate the acceptability and efficacy of UV/BL filtering masks and lenses for reducing ocular irradiance would be required.
5. Development of policies and practices for UV protection among water sport participants.

## Conclusion

The current absence of data on ocular UV and BL exposure in water sport participants should not be interpreted as evidence of negligible risk. Rather, it may highlight a gap in our understanding of environmental contributors to ocular disease. Given the intensifying effects of climate change and ozone fluctuation on UV exposure, individuals engaged in prolonged aquatic activities may face under-recognised ocular risks. We encourage research communities to systematically evaluate these issues, using best practice high-quality research approaches. Proactive investigation now may help to inform preventive strategies and mitigate long-term visual morbidity in these environmentally exposed groups.

Supplemental material is available at Eye’s website.

## Supplementary information

Appendix A

Appendix B
