# Supplementary material for: Are surfers and scuba divers an overlooked at-risk group for age-related macular degeneration?
Source: Eye (Lond). 2025 Jul 21;39(13):2495–6. doi: 10.1038/s41433-025-03941-9 (PMC12402220; doi:10.1038/s41433-025-03941-9)
Supplement: Supplementary file 1 — Appendix A [file 41433_2025_3941_MOESM1_ESM.docx]

# Appendix A – Search Strategy

(surf[Title/Abstract] OR surfer[Title/Abstract] OR surfing[Title/Abstract] OR "water sport*"[Title/Abstract] OR "outdoor sport*"[Title/Abstract] OR "ocean exposure"[Title/Abstract] OR fisher*[Title/Abstract] OR sailor*[Title/Abstract] OR “jet ski”[Title/Abstract] OR “wake board*”[Title/Abstract] OR “water ski*”[Title/Abstract]  OR "bodyboarding"[Title/Abstract] OR "stand-up paddleboarding"[Title/Abstract] OR "windsurfing"[Title/Abstract]  OR "kitesurfing"[Title/Abstract]  OR "kayaking"[Title/Abstract]  OR "canoeing"[Title/Abstract]  OR "open water swimming"[Title/Abstract]  OR "triathlon"[Title/Abstract]  OR "scuba diving"[Title/Abstract]  OR "snorkelling"[Title/Abstract] OR swimmer[Title/Abstract] OR kitesurfing[Title/Abstract] OR rowing[Title/Abstract])

AND

(ultraviolet[Title/Abstract] OR blue[Title/Abstract] OR “high energy visible” [Title/Abstract] OR sun[Title/Abstract] OR solar[Title/Abstract])

AND

(light[Title/Abstract] OR radiation[Title/Abstract])

AND

(macular[Title/Abstract] OR retina[Title/Abstract] OR retinal[Title/Abstract])

AND

(damage[Title/Abstract] OR degeneration[Title/Abstract] OR insult[Title/Abstract] OR injury[Title/Abstract])
