# Supplementary material for: Are surfers and scuba divers an overlooked at-risk group for age-related macular degeneration?
Source: Eye (Lond). 2025 Jul 21;39(13):2495–6. doi: 10.1038/s41433-025-03941-9 (PMC12402220; doi:10.1038/s41433-025-03941-9)
Supplement: Supplementary file 2 — Appendix B [file 41433_2025_3941_MOESM2_ESM.docx]

# Appendix B – PRISMA Flowchart

Records identified from:

EMBASE (n = 9)

PubMed (n = 6)

Scopus (n = 0)

SportDiscus (n = 0)

Web of Science (n = 7)

Records removed *before screening*:

Duplicate records removed (n = 10)

Records screened

(n = 23)

Records excluded

(n = 12)

Reports sought for retrieval

(n = 0)

Reports assessed for eligibility

(n = 0)

Studies included in review

(n = 0)

**Identification of studies via databases and registers**

**Identification**

**Screening**

**Included**
